# The Role of Osteoprotegerin in Breast Cancer: Genetic Variations, Tumorigenic Pathways, and Therapeutic Potential

**DOI:** 10.3390/cancers17030337

**Published:** 2025-01-21

**Authors:** Janan Husain Radhi, Ahmed Mohsen Abbas El-Hagrasy, Sayed Husain Almosawi, Abdullatif Alhashel, Alexandra E. Butler

**Affiliations:** 1 School of Medicine, Royal College of Surgeons in Ireland—Medical University of Bahrain (RCSI Bahrain), Building No. 2441, Road 2835, Busaiteen P.O. Box 15503, Bahrain; 21200402@rcsi-mub.com (J.H.R.); 20205713@rcsi-mub.com (A.M.A.E.-H.); 20204234@rcsi-mub.com (S.H.A.);; 2 Research Department, Royal College of Surgeons in Ireland—Medical University of Bahrain (RCSI Bahrain), Building No. 2441, Road 2835, Busaiteen P.O. Box 15503, Bahrain

**Keywords:** osteoprotegerin, breast cancer, single nucleotide polymorphisms, TRAIL, RANK, *BRCA1*

## Abstract

Introduction: Osteoprotegerin (OPG), encoded by the *TNFRSF11B* gene, is linked to the development of breast cancer via several pathways, including interactions with the receptor activator of nuclear factor-κB (RANK) ligands, apoptosis-inducing proteins like TRAIL, and genetic variations such as single nucleotide polymorphisms (SNPs), directly altering gene expression. This review aims to investigate the role of OPG expression in breast cancer. Methods: A comprehensive literature search was conducted using PubMed Medline, Google Scholar, and ScienceDirect. Only full-text English publications from inception to September 2024 were included. Results: Studies have demonstrated that certain SNPs in the OPG gene, specifically *rs3102735* and *rs2073618*, are linked to a higher risk of breast cancer development. Additionally, OPG’s function as a TRAIL decoy receptor may inhibit the death of cancer cells. Furthermore, OPG in the serum and its interactions with BRCA mutations are being investigated for their potential influence on breast cancer progression. Studies have found that OPG promotes tumorigenesis by enhancing cell proliferation, angiogenesis, and aneuploidy in normal mammary epithelial cells. Moreover, OPG mediates the tumor-promoting effects of interleukin-1 beta and may serve as a biomarker for breast cancer risk, particularly in BRCA1 mutation carriers, through its role in dysregulated RANK signaling. Lastly, the use of recombinant OPG in mouse models has been found to exert anti-tumor effects. Conclusions: In this review, the role of OPG in breast cancer is examined. OPG has a multifaceted role in breast cancer tumorigenesis and exerts its effects through genetic variations (SNPs), interactions with TNF-related apoptosis-inducing ligand (TRAIL), and the modulation of the pro-tumorigenic microenvironment effects of angiogenesis, cell survival, and metastasis. Additionally, OPG’s dual role as a tumor suppressor and promoter serves as a possible therapeutic target to enhance apoptosis, limit bone metastasis, and modulate the tumor microenvironment. Whilst much is now known, further studies are necessary to fully delineate the role of OPG.

## Simple Summary

This review investigates the role of osteoprotegerin (OPG) in breast cancer, exploring how genetic variations in the OPG gene (TNFRSF11B), its interactions with the RANK/RANKL pathway, and its modulation of cancer-related processes contribute to tumorigenesis. OPG’s dual role as both a tumor suppressor and promoter is highlighted, with its effects on cell survival, angiogenesis, and metastasis. Additionally, genetic mutations, such as those in BRCA1, influence the risk of breast cancer development in relation to OPG expression. The study underscores the potential of targeting the OPG/RANKL axis for therapeutic interventions, with a focus on enhancing apoptosis, reducing metastasis, and improving patient outcomes through personalized treatment strategies.

## 1. Introduction to Cancer

The overall prevalence of cancer in the United States is projected to rise significantly in 2024, with an estimated 2,001,140 new cancer diagnoses among both men and women. For females, approximately 972,060 new cases are expected across all cancer types. Breast cancer remains the most prevalent, accounting for 310,720 cases, making it the leading cancer diagnosis among women [1]. Breast cancer mortality decreased by 42% in 2021, primarily due to advancements in early detection through widespread mammography screening [1]. Despite this progress, breast cancer remains one of the leading causes of cancer-related death in women. These statistics highlight the importance of prognostic biomarkers for early detection of breast cancer. Prognostic biomarkers provide insights into tumor biology and enable earlier detection, patient stratification based on risk, and guidance for personalized treatment strategies.

Cancer therapy has evolved significantly over the years. Before the 1970s, it began with radiation therapy as a modality to treat cancer and evolved to chemotherapeutic agents such as nitrogen mustard gas, folate antagonists, and purine antimetabolites. These modalities demonstrated varying degrees of effectiveness depending on cancer type, with chemotherapy particularly effective against rapidly dividing malignant cells. Between the 1970s and 2023, new treatment approaches emerged, including hormonal therapies targeting specific mutations, such as trastuzumab, a monoclonal antibody that targets HER2 receptor-positive breast cells. Additionally, advancements like immune checkpoint inhibitors, oncolytic virus therapy, and chimeric antigen receptor (CAR) T-cell therapies have further revolutionized cancer treatment [2].

## 2. Introduction to Osteoprotegerin

**Figure 1:**
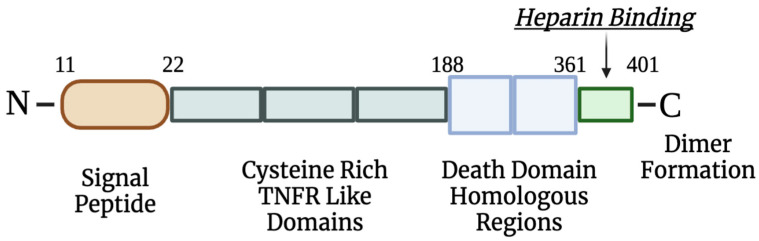
Osteoprotegerin (OPG) structure. OPG contains amino-terminal signal peptides and cysteine-rich tumor necrosis factor receptor (TNFR)-like domains, which are the key binding forces to the receptor activator of nuclear factor-κB (RANK). Additionally, OPG includes two death domain homologous regions of which the functions are currently not understood. A heparin binding domain limits the half-life of this molecule and serves as a basis for dimer formation [5].

Osteoprotegerin (OPG), a member of the tumor necrosis factor (TNF) receptor family, is a glycoprotein expressed by the *TNFRSF11B* gene and is secreted by stromal and osteoblastic cells [3]. Chromosome *8q23-24* encodes for OPG, which consists of 401 amino acids in the transmembrane form before it is cleaved to 380 amino acids, and its structure can be seen displayed below in Figure 1 [4]. OPG has various physiological functions in the body, but it mainly plays a role in the bone metabolism pathway by preventing the binding of the receptor activator of nuclear factor-κB (RANK) ligands to RANK, inhibiting osteoclast maturation and differentiation by binding to the receptor activator of nuclear factor-κB ligands (RANKL) [4].

OPG is important in maintaining normal bone physiology since it is involved in bone remodeling by inhibiting osteoclast activity through the RANKL-RANK pathway, thereby maintaining bone density and preventing conditions like osteoporosis. It is considered bone-protective due to its ability to suppress bone resorption and increase bone mass. Various cells in the bone marrow, including osteoblasts, B cells, megakaryocytes, platelets, vascular endothelial cells, and vascular smooth muscle cells, express and secrete OPG [4]. Beyond the skeleton, OPG is also expressed in tissues such as the heart, kidney, liver, and spleen. It also has been shown that OPG regulates vascular biology by interacting with tumor necrosis factor-related apoptosis-inducing ligand (TRAIL), preventing arterial calcification and atherosclerosis. Additionally, OPG modulates immunity by influencing B-cell development and regulatory T-cell function [4].

The RANK ligand, a type two transmembrane protein, is produced by osteoblasts and activated T-cells in its membrane form, and metalloproteinases cleave it to its soluble form [6]. The soluble form is neutralized by binding to OPG, which aids in maintaining a balance in bone metabolism by preventing excessive bone resorption through the binding of RANK/RANKL [7]. The interaction between RANKL and RANK on osteoclastic cells induces a signaling cascade that leads to the activation of transcription factors that are essential to bone resorption, such as nuclear factor kappa-light-chain-enhancer of activated B cells (NF-κB) [6]. RANK is expressed in various tissues and cells throughout the body, including osteoclasts, the mammary glands, and immune cells [8]. As a result, the interaction between RANK and RANKL serves multiple functions beyond bone remodeling, playing significant roles in mammary gland physiology and tumorigenesis [9].

NF-κB is a family of transcription factors that regulate many biological processes [10]. NF-κB controls genes expressed in immune responses and inflammation and is also involved in cellular proliferation by the induction of anti-apoptotic genes. These genes are crucial in cancer where NF-κB activity is dysregulated, leading to increased tumor cell survival [11].

**Figure 2:**
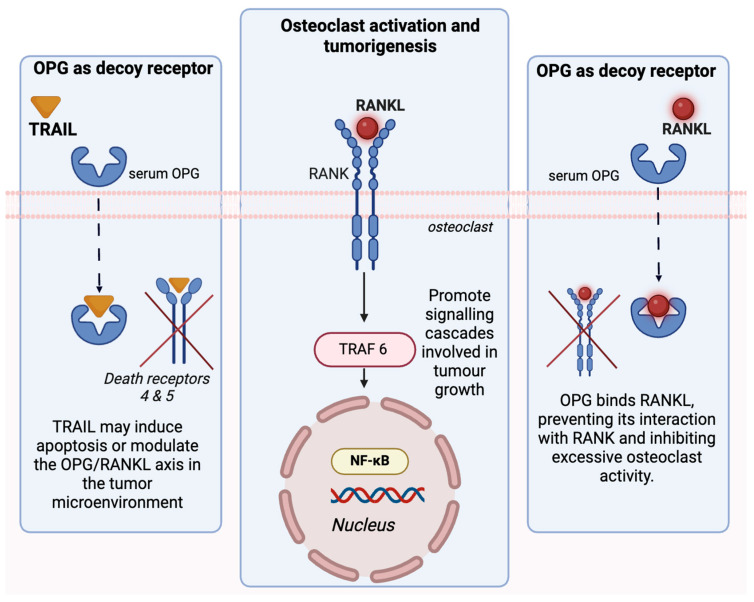
The OPG/RANK/RANKL pathway. A schematic to illustrate the OPG/RANK/RANKL pathway, which regulates bone remodeling by balancing osteoclast activation. The receptor activator of nuclear factor-κB ligand (RANKL) binds to the receptor activator of nuclear factor-κB (RANK) on osteoclast precursors, initiating a signaling cascade (via TRAF6 and NF-κB) that promotes bone resorption and is also involved in cellular proliferation. Osteoprotegerin (OPG) acts as a decoy receptor, binding RANKL to inhibit its interaction with RANK and prevent excessive osteoclast activity. Dysregulation of this pathway contributes to tumorigenesis. On the left, OPG binds to TNF-related apoptosis-inducing ligand (TRAIL), preventing its interaction with death receptors (DR4 and DR5) on tumor cells. This neutralization inhibits TRAIL-induced apoptosis, allowing cancer cells to survive.

Osteoprotegerin (OPG) exhibits a dual role in tumorigenesis, acting both as a tumor suppressor and promoter depending on the biological context. As a decoy for RANKL, OPG can inhibit RANK-mediated signaling, a pathway critical for osteoclast activation and, more broadly, for promoting tumor progression. Figure 2 displays the OPG/RANK/RANKL pathways, which regulates bone remodeling through the activation of osteoclasts. OPG exhibits tumor-promoting effects, as seen with reduced OPG levels; the unchecked RANKL activity contributes to bone metastases and tumor progression and survival. Conversely, OPG’s interaction with TNF-related apoptosis-inducing ligand (TRAIL) complicates its role in tumorigenesis. Acting as a decoy receptor for TRAIL, OPG prevents TRAIL-induced apoptosis, which inadvertently promotes tumor cell survival. Beyond its interaction with TRAIL, OPG may enhance tumor progression by fostering angiogenesis, modulating the tumor microenvironment, and aiding immune evasion [12].

Additionally, TNF-related apoptosis-inducing ligand (TRAIL) also binds to OPG [4]. TRAIL plays a significant role in programmed cell death by binding to the death receptors (DR4 & DR5) on the surface of targeted cells. Hence, it is crucial in the induction of apoptosis in selective cancer cells [13]. The expression of OPG has been found to increase cancers, such as lung and breast cancer, through the numerous interactions of OPG with other TNF receptor families [14].

## 3. OPG/RANK/RANKL Pathway & Cancer

An imbalance in the OPG/RANK/RANKL ratio can contribute to the progression of cancer, particularly cancer that metastasizes to the bone. According to prior studies, it has been established that RANKL is involved in the signaling of tumor initiation in cancers such as breast and prostate cancer through the activation of TNF receptor-associated factor 6 (TRAF 6), which further activates NF-κB [8]. Moreover, whether OPG plays a role in tumorigenesis has yet to be fully understood, as it exhibits antitumorigenic effects by acting as a decoy receptor for RANK; by contrast, its TRAIL-induced signaling is rendered tumor-promoting [15,16].

Bone metastasis progression occurs due to the formation of a favorable bone microenvironment that supports tumor cell survival [17]. This happens through the secretion of cytokines, growth factors, and hormones by cancer cells [16]. One significant hormone involved is parathyroid hormone-related protein (PTHrP), which plays an important role in altering the expression of RANKL and OPG [17]. In addition to PTHrP, tumor cells can directly express RANKL, leading to osteolysis, and secrete other pro-osteoclastogenic factors such as macrophage colony-stimulating factor (M-CSF), Interleukin (IL)-1α, IL-6, IL-8, and prostaglandin E2, all of which further promote bone degradation and support tumor growth within the bone microenvironment [16,17,18].

The RANKL-RANK-OPG system plays a critical role in osteoimmunology, particularly focusing on the interactions between bone and immune systems. OPG functions as a decoy receptor for RANKL, binding to it and preventing its interaction with RANK. This regulation is critical in modulating immune responses. By inhibiting RANKL, OPG can influence immune cell functions, including the differentiation and activity of regulatory T cells (Tregs). This modulation helps balance immune activation and tolerance, particularly in contexts such as autoimmune diseases and conditions involving bone destruction. The system’s broader relevance is noted in various diseases including metastatic bone tumors, where the balance of RANKL, RANK, and OPG plays an important role in disease progression and immune modulation [19].

While the interaction between OPG and RANKL has been extensively studied in bone metabolism and tumorigenesis, several gaps persist in our understanding of how this pathway operates in the tumor microenvironment. It is evident that excess RANKL activity and deprivation of OPG enhance osteoclast-mediated bone resorption and create a favorable microenvironment for tumor invasion and growth [20]. However, the role of OPG and RANKL expression within tumors requires further investigation, particularly in the context of breast cancer subtypes. Variations in expression levels may significantly impact tumor progression, angiogenesis, and immune cell infiltration, potentially shaping the tumor microenvironment. Exploring these dynamics could provide critical insights into the broader implications of the OPG-RANKL axis in cancer biology [8,12].

This review investigates the role of osteoprotegerin (OPG) in the pathogenesis of breast cancer, with a focus on understanding the effects of the RANKL/RANK/OPG pathway and single nucleotide polymorphisms (SNPs) in the OPG-encoding gene (*TNFRSF11B*). Additionally, this study aims to explore the potential impact of OPG on breast cancer risk in individuals carrying *BRCA1/2* mutations, to elucidate the underlying mechanisms contributing to breast tumorigenesis and progression.

## 4. Methodology

Various databases were used to allow for a high-quality literature search. Relevant articles were identified from inception until September 2024 in PubMed, Medline, Google Scholar, and ScienceDirect. The following keywords were used: osteoprotegerin (OPG), tumor, TNF-related apoptosis-inducing ligand (TRAIL), receptor activator of nuclear factor-κB (RANKL), prognostic marker, breast cancer gene (*BRCA*), therapeutic target, and breast cancer. Inclusion criteria included peer-reviewed articles published in English, with full-text availability, to ensure easier accessibility to studies, as most studies are published in English; however, this may have limited the scope of relevant studies, potentially overlooking regionally specific insights into OPG’s role in breast cancer. All types of studies, including preclinical trials, case-control studies, cohort studies, and reviews, were included. Studies were included if they specifically investigated any aspect with regard to the tumorigenesis of OPG, with a focus on tumorigenesis in breast cancer. Human studies and non-human experimental models, such as cell lines and animal models, that investigated OPG-related pathways in breast cancer were included. Exclusion criteria involved studies addressing OPG in diseases unrelated to breast cancer, non-English publications, and studies without full-text availability.

## 5. Breast Cancer

Breast cancer is a neoplastic transformation of normal breast cells to abnormal cancerous cells that develops in breast tissue. The uncontrollable growth of these abnormal cells in breast tissue causes the formation of a tumor in different compartments of the breast, commonly the ductal epithelium and breast lobules. A breast cancer is classified as invasive or non-invasive (in situ) breast cancer, depending upon whether the cancer cells have invaded into surrounding tissue or are confined to either the duct or lobule. Breast cancer is the most common cancer diagnosed in women, accounting for more than 1 in 10 new cancer diagnoses annually and is the second most common cause of cancer death among women worldwide [21]. Furthermore, breast cancer is the second most common cancer worldwide, accounting for an incidence of approximately 2.3 million new cases in 2022 alone among women [22]. Invasive breast cancer represented approximately 11.7% of new cases in 2020 [21]. In the United States, 1 in 8 women and 1 in 1000 men are expected to develop breast cancer at some point in their lives [21].

Breast cancer can be further classified based on histological type and location. The most common type of invasive breast cancer (50–75% of invasive breast cancers) is ductal adenocarcinoma, originating from the ducts, followed by lobular carcinoma (10–15%), mucinous carcinoma (also known as colloid carcinoma; 2–5%) and tubular carcinoma (1–2%) [21]. Additionally, a rare, aggressive subtype of invasive breast cancer, medullary carcinoma, is poorly differentiated and is the least common invasive breast cancer [21].

Risk factors for developing breast cancer include increasing age, female gender, previous history of breast cancer, histologic abnormalities on breast biopsy, family history of breast cancer, genetic mutations such as *BRCA1* and *BRCA2*, younger age at onset of menarche, nulliparity, menopause after the age of 55, exogenous hormone use such as hormone replacement therapy, and other external factors such as radiation and environmental exposure, excessive alcohol consumption, and obesity. Most cases of breast cancer are sporadic (90–95%) [21]. The strongest risk factor, however, remains increasing age in women, with the incidence rate increasing from 1.5 cases per 100,000 in women aged 20–24 years to a peak of 421.3 cases per 100,000 in women aged 75–79 years [21].

### 5.1. OPG in the Pathogenesis of Breast Cancer

Osteoprotegerin (OPG), encoded by the TNFRSF11B gene, has been shown to have a mechanistic role in breast tumorigenesis and progression. Although the mechanisms through which OPG plays a role in the pathogenesis of breast cancer are not fully delineated, risk factors include single nucleotide polymorphisms (SNPs) in the OPG gene, effects on the TNF-related apoptosis-inducing ligand, OPG protein levels, the interplay of OPG protein levels with *BRCA* mutations, and the overexpression of certain receptors [14].

Interestingly, the promoter region of the TNFRSF11B gene itself can undergo hypermethylation. As previously discussed, this gene serves as a negative regulator of RANKL-mediated signaling by binding to RANK. During hypermethylation, transcriptional changes occur on the gene, subsequently leading to gene silencing and OPG downregulation, and ultimately to reduced RANKL neutralization. This epigenetic dysregulation disrupts the RANK/RANKL/OPG axis, shifting the balance towards RANK/RANKL interactions, which leads to increased osteoclastogenesis and bone metastasis [23].

### 5.2. Single Nucleotide Polymorphisms (SNPs) of the OPG Gene & Breast Cancer

SNPs of *TNFRSF11B*, the OPG-encoding gene located on chromosome *8q23-24*, have been associated with an increased risk for development of breast cancer. Three main SNPs have been the focus of studies investigating the association of OPG with developing breast cancer: *rs3102735*, *rs2073617*, and *rs2073618* [14]. A study investigating the association between the frequency of two OPG gene SNP mutations, *rs3102735* and *rs2073618*, in over 1400 women, 614 of which had breast cancer, revealed that the minor C allele of SNP *rs3102735* was associated with a 1.5-fold increased risk for breast cancer tumorigenesis [14]. The increased risk from the SNP of *rs3102735* may be explained through its effect on altering transcription factor binding in the promoter region, leading to altered expression of the *TNFRSF11B* gene. Any increased expression or dysregulation of OPG expression, caused by alterations to its encoding gene, *TNFRSF11B*, may influence the OPG/RANK/RANKL axis, which may explain the increased breast cancer risk with this polymorphism. Although no association was found between the SNP *rs2073618* and risk of breast cancer, the study established that the presence of the major allele G increased the likelihood of invasive breast cancer [14].

A similar study investigating the increase in likelihood of developing breast cancer in the presence of OPG SNPs found that the SNP *rs2073618* minor allele C was associated with an increased frequency of breast cancer [14]. Further, this same study established a higher frequency of the major T allele in the OPG gene SNP *rs2073617* in breast cancer. Conversely, a combined genotype heterozygous for the GG major allele for OPG *rs2073618* and the CC minor allele for OPG *rs2073617* was found to be protective against the development of breast cancer [14].

The disease-free period and prognosis of breast cancer patients may also be influenced by OPG SNPs. In a study by Ciscar et al., patients with the SNP *rs34945627* had reduced disease-free survival periods, as well as decreased overall survival [24]. In a study investigating the association of RANKL and OPG polymorphisms with Aromatase Inhibitor-Related Musculoskeletal Adverse Events (AI-related MS-AEs) in Chinese breast cancer patients, the SNPs *rs7984870* of RANKL and *rs2073618* of OPG were associated with development of AI-related MS-AEs [25].

Collectively, these studies highlight the somewhat under-investigated role that single nucleotide polymorphisms (SNPs) of the OPG gene play in the pathogenesis of breast cancer. Table 1 below displays the SNPs in the OPG gene along with some of the therapeutic opportunities that they may offer.

### 5.3. OPG and TNF Related Apoptosis-Inducing Ligand (TRAIL)

TRAIL induces apoptosis in cancer cells, and OPG (osteoprotegerin) can act as a decoy receptor for TRAIL, potentially inhibiting its pro-apoptotic effects and thus contributing to cell survival in breast cancer [20,26]. In a study by Holen et al., OPG was shown to be an endocrine survival factor in MDA-MB-436 and MDA-MB-231 human breast cancer cells by significantly reducing TRAIL-induced apoptosis in vitro [27]. Thus, breast cancer cells may use OPG expression to gain survival advantages over host defences. By contrast, in vivo, no effect of OPG on TRAIL was demonstrated, even with supraphysiological concentrations of OPG, which may be explained by the simultaneous presence of RANKL and TRAIL [28]. Moreover, the presence of excess RANKL in vivo can reverse the effects of OPG on TRAIL-induced apoptosis in human breast cancer cells, even though OPG binds to TRAIL and RANKL with the same affinity as in vitro [28]. Thus, direct proliferative action of OPG on primary human breast cancer cells through reduction in TRAIL-induced apoptosis is a potential mechanism underlying breast tumorigenesis in vitro, though this requires confirmation in further in vivo studies.

### 5.4. Indirect Tumor-Promoting Effects of OPG

Beyond the direct proliferative actions of OPG on breast cancer cells and in breast tumorigenesis, studies have investigated other mechanisms whereby OPG may exert indirect tumor-promoting effects towards the development of breast cancer [20]. One theory suggests that OPG promotes tumor growth through pro-angiogenic effects. Cross et al. found a positive association between endothelial OPG expression and high tumor grade, while Goswami et al. demonstrated OPG’s role in driving neoangiogenesis, potentially by promoting endothelial cell survival and differentiation and thereby stimulating breast tumor growth [29,30]. Another proposed mechanism is that OPG reprograms normal mammary epithelial cells into a tumorigenic state by inducing proliferation and aneuploidy, further contributing to cancer development [30].

OPG has also been shown to enhance breast cancer invasion and metastasis. Weichhaus et al. demonstrated that OPG modulates the expression of proteases such as cathepsin D and matrix metalloproteinase-2, facilitating cancer cell metastasis [31]. The authors also showed that knockdown of OPG in TNBC cells leads to a significant reduction in metastasis in the chick embryo metastasis model [31].

Thus, OPG’s effects in breast tumorigenesis may not be limited to direct effects but also indirect tumor-promoting effects, including pro-angiogenic effects, promoting endothelial cell survival and differentiation, and inducing proliferation.

### 5.5. OPG Protein Serum Levels & Breast Cancer Subtypes

The relationship between OPG protein levels in serum and risk for development of breast cancer was investigated. OPG protein serum levels were found to have a contrasting role in breast cancer tumorigenesis based on the breast cancer subtype, namely ER-positive versus ER-negative breast cancer. In the Tromsø study by Vik et al., serum samples from 76 subjects in Norway that developed breast cancer were categorized into tertiles for serum levels of OPG: 0.46–2.78 ng/mL, 2.79–3.55 ng/mL, and 3.56–25.81 ng/mL, respectively [32]. Women in the upper tertile of OPG had a 45% reduced risk of breast cancer compared to the lowest tertile [32]. Additionally, a 76% relative risk reduction was observed with high levels of OPG in the subset of women <60 years.

The European Prospective Investigation into Cancer and Nutrition (EPIC) cohort study found that women with higher OPG serum levels had an increased relative risk (1.93) of developing estrogen receptor-negative (ER–) breast cancer [33]. This risk was observed across tertiles of OPG serum levels, with higher levels correlating with more ER− cancer cases [33]. A modest risk reduction of 0.84 for patients with ER+ breast cancer was observed with high levels of OPG [33].

Although the Vik et al. study did not stratify the risk of breast cancer relative to OPG levels according to ER status, it is likely that both studies are in agreement that low OPG serum levels are associated with reduced risk of ER+ breast cancer, whilst higher OPG serum levels are associated with increased risk of ER− breast cancer. Thus, the likelihood of the development of either ER+ or ER− breast cancer subtypes may be associated with OPG serum levels.

Overall, the presence of high OPG levels may increase the risk of ER-negative breast cancer while being protective against ER-positive breast cancer cases. The protective effect may stem from the antagonizing role that OPG plays in RANK signaling, which is crucial for mammary epithelial cell development and proliferation [7,32]. By preventing the RANK/RANKL interaction as OPG binds to RANKL, the potential pro-proliferative effects of the RANK/RANK axis are limited in ER-positive cells. This provides a therapeutic opportunity for OPG or other analogues as preventative agents in at-risk individuals with hereditary or familial hormone-sensitive breast cancers. Additionally, combining their use with RANKL inhibitors such as denosumab may mitigate the risk of RANKL-driven tumorigenesis in ER-positive cases [34]. On the other hand, the association of increased risk of ER-negative breast cancer with high OPG levels may be linked to OPG’s role as a decoy receptor for TRAIL, and therefore inhibiting TRAIL-induced apoptotic effects in neoplastic cells and enhancing the survival of ER-negative tumor cells [33,35]. Therefore, a number of clinical opportunities arise that can be implemented to capitalize on the treatment of ER-negative breast cancer cases with high OPG levels, including targeting the OPG–TRAIL interaction through the development of TRAIL receptor agonists or inhibitors of OPG–TRAIL binding. A TRAIL receptor agonist would restore apoptosis in tumor cells and counteract tumor progression, particularly in aggressive cases such as triple-negative breast cancer (TNBC).

### 5.6. OPG and Breast Cancer Risk with BRCA Gene Mutations

The ability of OPG to block RANKL activity has raised the question as to potential underlying mechanisms in which OPG may play a role in the development of *BRCA* mutation breast cancer. OPG’s role in the development of *BRCA1*-mutated breast cancer is due to the known effects that RANKL plays in breast cancer development in patients with *BRCA1* mutations [14,36]. In a study investigating serum OPG levels in 391 *BRCA1/2* mutation carriers and 782 non-carrier healthy subjects, *BRCA* mutation carriers had significantly lower OPG levels compared to healthy subjects. However, this study did not follow up on whether the lower OPG levels were associated with an increased risk of breast cancer, leaving the relationship between OPG and cancer development unclear [37].

In a subsequent study, serum OPG levels in 206 women with *BRCA1/2* mutations were followed up for 6.5 years [37]. Women with lower baseline OPG levels were more likely to develop breast cancer (13 of 103) compared to those with higher OPG levels (6 of 103). Although the study showed an association between lower OPG levels and breast cancer development, the results were only marginally significant due to the small sample size.

**Figure 3:**
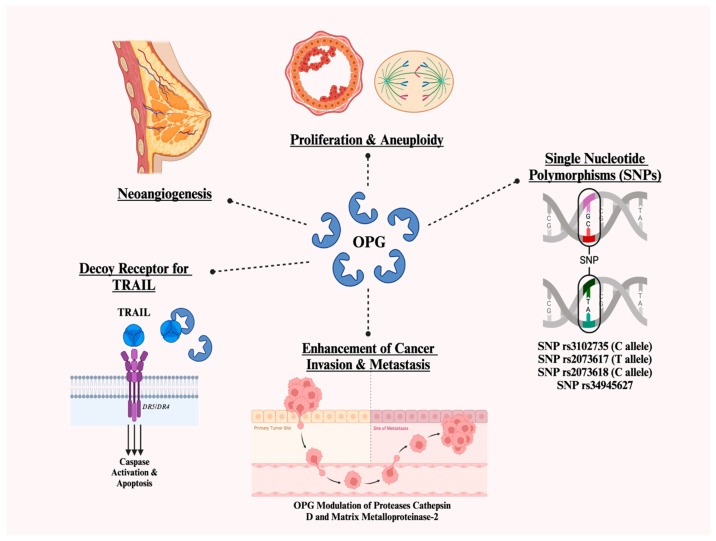
Osteoprotegerin (OPG)-mediated mechanisms in cancer progression. OPG enhances proliferation and promotes aneuploidy by activating chromosomal instability pathways involving Aurora A, Bub1, and BubR1 kinases, resulting in genomic disruption. Moreover, OPG drives neoangiogenesis by enhancing endothelial cell survival and vascular network formation through paracrine signaling, and facilitating tumor growth and progression, which is further reinforced by OPG’s ability to stimulate endothelial cell survival and differentiation. Moreover, OPG serves as a decoy receptor for tumor necrosis factor-related apoptosis-inducing ligand (TRAIL), which prevents apoptosis. OPG also mediates protease activity, including cathepsin D and matrix metalloproteinase-2 (MMP-2), to facilitate cancer invasion and metastasis. Furthermore, single nucleotide polymorphisms (SNPs) associated with OPG (e.g., *rs3102735, rs2073617, rs2073618, rs4845627*) may influence susceptibility to malignancy.

Though the limited available studies do not clearly establish a link between OPG serum levels and the risk of breast cancer in *BRCA* mutation carriers, these findings suggest a potential mechanism that warrants further investigation, particularly given the elevated breast cancer risk in the *BRCA* carrier patient population. A summary of the implicated OPG-mediated mechanisms in cancer progression discussed are shown below in Figure 3.

## 6. Role of OPG in Promoting Cancers

### 6.1. Proliferation and Angiogenesis

OPG plays a pivotal role in inducing aneuploidy, angiogenesis, and cell proliferation within the breast cancer microenvironment [30]. It functions as both an endocrine and paracrine signaling molecule, potentially reprogramming normal mammary epithelial cells into a tumorigenic state [30]. In a series of experiments using in vitro sphere cultures of human mammary epithelial cells (HMEC), researchers depleted OPG from conditioned media derived from breast cancer lines (*SUM149PT* and *SUM1315MO2*), achieving high depletion rates [30].

The role of OPG in promoting angiogenesis was further explored through tube formation assays with HMVEC-d endothelial cells. Results indicated that OPG-rich media facilitated the formation of complex, dense networks of tubes characterized by increased length, width, and branch points compared to control media [30]. Conversely, OPG-depleted media produced poorly defined and primitive tube structures, indicating OPG’s importance in vascular development [30]. The addition of recombinant human OPG restored the formation of intricate tube networks [30].

In addition to its angiogenic effects, OPG significantly influenced the proliferation of HMEC spheres [30]. Observations showed that spheres cultured in OPG-rich conditioned media were larger, more numerous, and denser compared to those in control media [30]. This effect was markedly diminished in OPG-depleted media, emphasizing OPG’s role as a critical survival factor for these cells. When recombinant OPG was re-introduced, the proliferation of the spheres was increased, confirming OPG’s vital role in tumor growth and survival [30].

### 6.2. Aneuploidy

OPG has been identified as a significant factor in inducing aneuploidy in normal HMEC spheres, a hallmark frequently associated with cancer progression [30]. Research indicates that the presence of OPG in the breast cancer microenvironment plays a critical role in this process [30]. Various studies have analyzed HMEC spheres grown in different media types, including standard HMEC media, conditioned media from breast cancer cell lines *SUM149PT* and *SUM1315MO2*, OPG-depleted conditioned media, and HMEC media supplemented with recombinant human OPG at concentrations of 500 pg/mL and 1100 pg/mL.

Propidium iodide (PI) staining and cell cycle analysis have shown that HMEC spheres in standard media remain entirely diploid, indicating stable genomic integrity [30]. However, exposure to conditioned media from *SUM149PT* or *SUM1315MO2* cells leads to a distinct shift, resulting in an increased population of aneuploid cells with a corresponding decrease in diploid cells [30]. By contrast, OPG-depleted media significantly reduces the occurrence of aneuploidy, underscoring the impact of OPG on chromosomal stability. Interestingly, the introduction of recombinant OPG also results in an increased incidence of aneuploidy in the HMEC spheres, reinforcing its role as a key contributor to genomic alterations [30].

**Figure 4:**
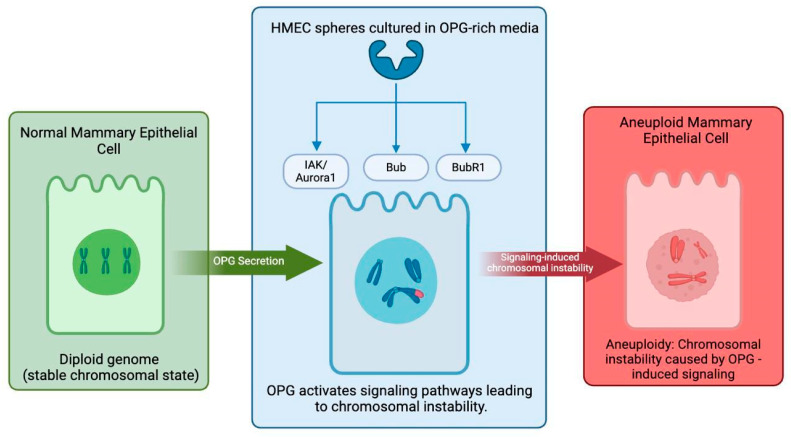
The role of osteoprotegerin (OPG) in inducing aneuploidy in mammary epithelial cells. A schematic to portray how osteoprotegerin (OPG) leads to chromosomal instability in human mammary epithelial cells (HMECs). The left panel represents a typical HMEC with a diploid genome and stable chromosomes. The middle panel shows the changes in the chromosomal integrity after the secretion of OPG in the tumor microenvironment. OPG has been shown to upregulate aneuploidy-related kinases such as Aurora A kinase (IAK-1), Bub1 kinase, and BubR1 protein, which disrupt mitotic checkpoint integrity and genomic stability. These regulatory effects underscore OPG’s contribution to driving aneuploidy and tumorigenesis. The right panel illustrates an aneuploid HMEC with disorganized chromosomes, highlighting the role of OPG in promoting chromosomal instability and aneuploidy, a key feature in cancer progression.

Further investigations into the mechanisms driving OPG-induced aneuploidy have focused on specific aneuploidy-related kinases, including Increase-in-Ploidy and Aurora-related Kinase 1 (IAK-1) with Aurora A, shortly referred to as IAK-1/Aurora A, Budding Uninhibited by Benzimidazoles 1 (Bub1) kinase, and Budding Uninhibited by Benzimidazoles-Related 1 (BubR1) protein. These kinases are found to be upregulated in breast cancer spheres compared to normal HMEC spheres [30]. Additionally, HMEC spheres cultured in OPG-rich media exhibit elevated levels of these kinases, supporting the hypothesis that OPG adversely affects the genomic integrity of otherwise healthy HMEC cells, thus facilitating the onset of aneuploidy [30]. Collectively, these findings suggest that OPG secreted by inflammatory and invasive breast cancer cells not only promotes cell proliferation but also significantly contributes to the development of chromosomal abnormalities [30]. Figure 4 below summarizes the role that OPG plays in inducing aneuploidy in breast epithelial cells.

### 6.3. Role of Osteoprotegerin in Interleukin-1 Beta-Induced Tumorigenesis of Breast Cancer Cells

IL-1B is a pro-inflammatory cytokine and plays a major role in the tumor environment to ensure cancer cell survival and growth [38]. The main mechanism underlying IL-1B-induced OPG involves the activation of the p38 and p42/44 MAPK signaling pathways, which result in a further downstream signaling cascade. Treatment with IL-1B leads to enhanced phosphorylation of these pathways, which are known to regulate gene expression, proliferation, and survival, while inhibition of p38 and p42/44 effectively reduces OPG secretion [39]. Notably, dual inhibition of both pathways resulted in even lower OPG levels in one study, suggesting their essential role in mediating IL-1B’s effects [39]. After the resultant increase in OPG levels, TRAIL-induced apoptosis is inhibited, thereby promoting tumor cell survival [4]. Additionally, OPG has been implicated in promoting the invasiveness of breast cancer cells, where continued increased OPG expression contributes to neoangiogenesis and metastasis through supporting endothelial cell survival and protease activity [39]. Evidence shows that IL-1B boosts the invasive capabilities of triple-negative breast cancer (TNBC) cells, and knocking down OPG expression diminishes this effect [39]. Thus, OPG acts as a crucial mediator in the tumor-promoting actions of IL-1B in breast cancer [39].

Interleukin-1 beta (IL-1B) has been shown to induce OPG expression in various breast cancer subtypes [39]. In triple-negative breast cancer (TNBC) cell lines, such as MDA-MB-436, MDA-MB-231, and BT549, higher basal levels of OPG and IL-1B were detected compared to non-TNBC lines like T47D and SKBR3 [39]. Notably, IL-1B significantly increased both OPG mRNA and protein levels across all cell lines tested, particularly in those with initially low OPG levels, indicating a strong response to IL-1B [39]. The IL-1B-induced upregulation of OPG has far-reaching implications in cancer biology, where it highlights its immune evasion functions by allowing tumor cells to evade immune surveillance by the blocking of TRAIL by OPG, and further portrays the microenvironment support that IL-1B provides by recruiting inflammatory cells, promoting, neoangiogenesis, and enhancing stromal support through its role as a pro-inflammatory cytokine [39]. IL-1B only constitutes one of the many immunomodulatory mechanisms at play in the breast cancer microenvironment. Recent studies have also highlighted how cancer cells reshape their environment to evade immune responses, allowing for further tumor growth and progression [40,41]. One of the ways that tumor cells downregulate the immune response is through inducing T cell exhaustion by upregulation of inhibitory receptors and changing cytokine profiles [40]. These exhausted CD8+ T cells are characterized by their impaired effector functions and reduced responsiveness to detected foreign antigens [40]. Another mechanism to improve breast cancer survival is through macrophage polarization, where macrophages are skewed to the alternatively activated macrophage (M2) phenotype through cytokines including IL-10 and IL-1B, resulting in increased angiogenesis, extracellular matrix remodeling, and, importantly, immune suppression [41].

### 6.4. Impact of RANK Signaling Pathway on BRCA-1 Associated Breast Cancer Development

OPG, a decoy receptor for RANKL, is found at lower levels in *BRCA1* mutation carriers, suggesting its potential as a biomarker for breast cancer risk [14,42]. This reduction in OPG may contribute to dysregulated RANK signaling, which is implicated in mammary tumorigenesis, especially in response to progesterone [42]. Evidence indicates that RANK signaling promotes the proliferation of mammary epithelial cells, thus enhancing cancer risk [14,42].

Further, research has highlighted the connection between elevated progesterone levels and upregulation of the RANK pathway, with implications for breast cancer development in women with *BRCA1* mutations [42]. Hormone replacement therapy (HRT), particularly progesterone-containing therapy, has been linked to an increased risk of breast cancer in these women, reinforcing the importance of RANK signaling in this context [14,42].

## 7. Anti-Tumor Properties of OPG

### 7.1. Key Molecular Pathways in Breast Cancer Tumorigenesis: RANKL/RANK and β-Catenin Signaling

Tumorigenesis in breast cancer is a multifaceted process that involves several molecular pathways. Among these pathways, the RANKL/RANK and B-catenin pathways play an important role in the regulation of cell proliferation [43]. The RANKL/RANK pathway regulates the proliferation of mammary epithelial cells [18]. However, because NFkB is a crucial regulator of the immune response and cellular proliferation, any imbalance in this interaction may promote the development of breast cancer.

Another pathway is B-catenin, which is pivotal in regulating cell proliferation and differentiation. B-catenin signaling with a wingless-related integration site (wnt) is involved in carcinogenesis and is also associated with tumor progression and metastasis [44]. Interestingly, OPG has been shown to inhibit the β-catenin pathway in breast cancer cells, potentially offering a therapeutic target for controlling tumor growth.

### 7.2. Protective Role of Stromal Fibroblasts in Breast Tumorigenesis

Normal breast tissue contains stromal fibroblasts, which are essential for maintaining the structural integrity and architecture of the organ. These fibroblasts play a protective role in carcinogenesis by inhibiting the growth of normal epithelium [45]. Moreover, the secretion of OPG by these fibroblasts inhibits several key breast-related signaling pathways, such as the nuclear factor kappa-light-chain-enhancer of activated B cells (NFkB). Inhibition of the interleukin 6 (IL-6)/signal transducer and activator of transcription 3 (STAT3) epigenetic feedback loop by OPG prevents the maintenance of epithelial-mesenchymal transition (EMT) and stemness features in breast cancer cells, which are essential for cancer progression and metastasis. However, studies have shown that fibroblasts derived from breast cancer cells exhibit less growth inhibitory capacity and actually enhance epithelial growth. In cancerous cells, OPG function has been linked to promoting tumorigenesis [45].

### 7.3. Recombinant OPG in Mouse Models

Recombinant osteoprotegerin (rOPG) is a laboratory-engineered version of the normally occurring OPG and is formed by genetic recombination. rOPG has significant antitumor effects in breast cancer through several mechanisms and the use of mouse models and a variety of breast cancer cell lines, including MDA-MB-231, MCF-7 and BT-20, has provided evidence supporting these effects. When these cells were injected with 0.1 μg/mL of rOPG, an inhibitory effect on important signaling pathways was induced [46].

One of the key pathways affected is the B-catenin/wnt pathway, which is important in maintaining the stemness and invasiveness of breast cancer stem cells [47]. Overall, these findings suggest that rOPG has the potential to be an effective therapeutic agent in targeting breast cancer stem cells, particularly by modulating the β-catenin/wnt signaling pathway by reducing the expression of the stemness markers which are essential for self-renewal of cancer cells.

Another report utilized a different cell line from a mouse model, 4T1.2Luc, alongside MDA-MB 231 and MCF-7. Similarly to human breast cell lines, 4T1.2Luc has been found to express RANK, thus providing a model for testing the anti-tumor effects of OPG. The role of the RANKL/RANK pathway in breast tumorigenesis is well established; the addition of RANKL to the cell line increased proliferation, whereas the addition of OPG plus RANKL decreased the growth rate of 4T1.2Luc cells to baseline levels [48]. Additionally, the effect of rOPG on bone metastasis has been tested in five different mouse models of breast cancer; inhibition of skeletal metastases occurred when given therapeutically or as a preventative measure [9].

## 8. OPG as a Biomarker

Osteoprotegerin (OPG) is emerging as a potential biomarker for breast cancer, particularly in relation to disease progression and prognosis. A study assessing overall survival (OS) and disease-free survival (DFS) revealed that high OPG levels, along with elevated RANKL and progesterone, significantly increase the risk of breast cancer [49]. Additionally, a trend has been observed linking RANKL expression to a more favorable prognosis for OS, suggesting that the RANKL/OPG pathway may play a crucial role in risk prediction for breast cancer patients. These findings highlight the pathway’s potential for improving current risk models and targeted prevention strategies by stratification of patients with high risk of progression. However, further investigation is still needed to establish whether these serum markers can be considered prognostic factors.

### Levels of Circulating OPG in Different Breast Cancer Subtypes

The EPIC study revealed that ER− breast cancer incidence is higher among individuals with elevated serum OPG levels (*p*-value 0.003) [33]. Similarly, another study confirmed that OPG could serve as a biomarker, particularly for ER− breast cancer, as elevated circulating OPG levels have been observed in this subtype. By contrast, no such increase has been detected in ER+ breast cancer [27]. In an EPIC cohort, Sarnik et al. reported that ER+ breast cancer is associated with increased levels of RANKL, rather than OPG, emphasizing different biomarker profiles for breast cancer subtypes [50].

Circulating OPG levels could be beneficial in identifying at-risk patients. One study identified a 5.33-fold higher risk of developing breast cancer among women with a high ratio of serum RANKL/OPG [42]. Similarly, Kiechl et al. found a five-fold increase in breast cancer risk among postmenopausal women with elevated progesterone and RANKL levels, in parallel with changes in the RANK/OPG ratio [50].

Moreover, in individuals with inherited *BRCA1* and *BRCA2* mutations, circulating serum OPG levels have been found to be lower. This suggests a potential link between OPG levels and genetic predisposition to breast cancer, highlighting its relevance as a biomarker in high-risk populations. Conversely, those with *BRCA1/2* mutations and high serum OPG levels have been found to have a significantly decreased risk of breast cancer [51].

## 9. Therapeutic Opportunities Derived from Osteoprotegerin Pathway Modulations in Breast Cancer

The investigation into the OPG modulatory pathways that can exert alterations in the tumorigenesis of breast cancer in this review allows for the exploration of potential therapeutic opportunities in the treatment of breast cancer.

### 9.1. Targeting the OPG/TRAIL Interaction

Firstly, the OPG/TRAIL interaction can be targeted, as OPG has been shown to inhibit TRAIL-induced apoptosis of Jurkat cells, known as immortalized human T lymphocytes [26]. Therefore, focusing on the development of agents that block the OPG protein from acting as a decoy receptor for the TRAIL receptor, and therefore disrupt the binding of OPG to TRAIL, can allow TRAIL to induce apoptosis in breast cancer cells effectively [27]. These include TRAIL receptor agonists, including agents such as dulanermin, which has been shown to have little activity in killing cancer cells alone. However, when combined with other synergistic drugs, it has demonstrated its increased activity in killing cancer cell lines, including ovarian and lung cancer [52]. Therefore, combination treatment incorporating dulanermin with other synergistic agents may provide an effective therapeutic potential in the treatment of breast cancer, where TRAIL’s pro-apoptotic effects can be restored, and the agents can be used to selectively induce cancer cell death.

Although dulanermin’s efficacy has only been explored in preclinical studies, its usage in human clinical trials appears feasible given that the challenges with its implementation are addressed. Firstly, the drug delivery method needs to be addressed, as systemic TRAIL activation may also affect healthy non-neoplastic cells, resulting in adverse effects or other pharmacokinetic effects such as rapid serum clearance [53,54]. Therefore, delivery through targeted delivery methods such as nanoparticles or tumor-specific ligands will prove vital for its effectiveness and success. Next, the presence of both RANKL and TRAIL simultaneously in vivo may counteract the effects of OPG–TRAIL- targeted therapies. Thus, addressing the factors that lead to this complex tumor microenvironment will be critical to ensure the success of TRAIL-based therapies [55]. Previous studies, including a study conducted by Li et al., have demonstrated how xenograft models can mimic the tumor microenvironment, simulating both tumor progression and metastatic pathways and providing a robust platform to allow for testing targeted interventions such as TRAIL-based therapies [56]. The mouse model used in the study was shown to successfully simulate the effects of different anesthetic agents on breast cancer development, deepening our understanding of breast cancer progression in the xenograft model [56]. Thus, future studies should capitalize on this opportunity to incorporate patient-derived xenograft models when testing the efficacy of TRAIL-therapies in disrupting the OPG/TRAIL interaction in tumor microenvironments that closely resemble human breast cancer. Other pre-clinical combination therapies can also be tested on this xenograft model, where large-scale clinical studies can investigate the effectiveness of therapies such as combined TRAIL receptor agonists alongside RANKL inhibitors to assess the optimal therapeutic regimens in the treatment of breast cancer.

### 9.2. Modulating the OPG/RANK/RANKL Pathway & Recombinant OPG (rOPG)

The OPG/RANK/RANKL pathway also offers a potential target for breast cancer therapy. RANKL inhibitors can be integrated into a multidisciplinary treatment to disrupt the pro-tumorigenic effects mediated by the OPG/RANK/RANKL axis. RANKL inhibitors can be used to prevent the progression of breast cancers, especially in cases of bone metastases, where denosumab, an FDA-approved RANKL inhibitor, is already in use to treat bony metastases from malignancies [57]. Denosumab is a monoclonal antibody that targets the RANK ligand and inhibits osteoclast activity, which may prove beneficial in breast cancer cases demonstrating dysregulated OPG/RANK/RANKL activity [58]. Denosumab is administered subcutaneously and, in the setting of breast cancer, may play a role in both diminishing bone loss from antihormonal treatments and preventing skeletal-related complications in metastatic breast cancer [58]. Additionally, rOPG has been investigated in preclinical studies on murine models and has been demonstrated to have a dual effect of reducing bone metastases and inhibiting breast cancer stem cell signaling through β-catenin/wnt pathways [59,60,61]. This provides the opportunity for exploring rOPG’s potential as a dual-action therapeutic in the treatment of breast cancer, where its mechanism of action involves blocking RANKL to reduce bone metastasis in breast cancer while also reducing the progression of breast cancer through targeting the β-catenin/wnt pathway, inducing inhibition of cancer stem cell signaling.

In *BRCA1* mutation carriers, there is an increased risk for the development of breast cancer, conferred by the functional inactivation of the tumor suppressor role that the *BRCA1* gene plays. This mutation subsequently results in reduced or dysfunctional DNA repair and gene transcription regulation, leading to the tumorigenesis of many cancers, including breast cancer [62,63,64,65,66]. This presents an opportunity for further combination therapy in breast cancer targeting both RANKL and progesterone in *BRCA1* mutation carriers. RANKL inhibitors such as denosumab may be combined with hormone modulators such as progesterone antagonists or even inhibitors of RANK signaling, which may reduce mammary epithelial proliferation and counteract the elevated breast cancer development risk involved in *BRCA1* mutation carriers [67].

### 9.3. OPG-Targeted Antibody Therapy, Anti-Angiogenesis Approaches, & SNP-Based Personalized Therapy

Potential therapies to combat OPG’s effects on promoting breast tumorigenesis may include OPG-targeted antibody therapy. Goswami et al.’s study has highlighted the role that OPG plays in promoting breast tumor proliferation and angiogenesis in invasive breast cancer [30]. Thus, the development of monoclonal antibodies to neutralize the effects of OPG activity in tumors can prove effective, thereby reducing its tumor-promoting effects in breast cancer. Preclinical studies have previously explored the effects of anti-OPG antibodies in murine models, highlighting the role that anti-OPG antibodies can play in developing pulmonary arterial hypertension (PAH) through promoting cell survival, pro-proliferative, and pro-migratory signaling. By administering a human anti-OPG therapeutic antibody (Ky3) in murine models, PAH was significantly reduced and reversed by diminishing pulmonary vascular remodeling in small pulmonary arterioles [68]. This anti-angiogenic effect may also prove beneficial when combined with other anti-angiogenic approaches such as the use of bevacizumab, an anti-vascular endothelial growth factor (anti-VEGF) antibody drug, that has shown efficacy in disrupting tumor vascularization in breast cancer [69]. These anti-angiogenesis approaches, combined with direct anti-OPG antibody targeting, can be combined with existing chemotherapies or checkpoint inhibitors routinely used in the treatment of breast cancer for a synergistic therapeutic effect.

Lastly, this review has identified the presence of specific SNPs in the OPG gene, such as rs3102735 or rs2073618, which are associated with an increased risk of breast cancer, providing the opportunity for SNP-based personalized therapy [14]. Patients with specific SNP mutations can be screened and identified early in the breast cancer treatment journey to allow their stratification for therapies targeting the OPG/RANKL pathway.

## 10. Conclusions

In conclusion, this review explores the role of osteoprotegerin in breast cancer pathogenesis. OPG has a multifaceted role in breast cancer tumorigenesis and exerts its effects through genetic variations (SNPs), interactions with TNF-related apoptosis-inducing ligand (TRAIL), and modulation of pro-tumorigenic microenvironment effects of angiogenesis, cell survival and metastasis. These findings leverage the mechanisms and pathways identified and propose actionable therapeutic opportunities that can be applied in treating breast cancer. Future research should further elucidate OPG’s role and utility as a biomarker and therapeutic target.

## Figures and Tables

**Table 1:** SNPs in the OPG gene and their therapeutic implications. A summary of key single nucleotide polymorphisms (SNPs) in the OPG gene and their therapeutic implications. Each SNP is associated with specific genetic effects and clinical outcomes, which can influence breast cancer risk, treatment response, and prognosis.

SNP	Effect	Therapeutic Implications	Study
*rs3102735*	Minor C allele associated with a 1.5-fold increased risk of breast cancer tumorigenesis.	Screening for this SNP can help identify high-risk individuals for targeted prevention and early intervention.	Geerts et al. [14]
*rs2073618*	Minor allele C is linked to increased breast cancer frequency, but the GG major allele was found to be protective against breast cancer. This SNP in OPG has also been associated with the development of Aromatase Inhibitor-Related Musculoskeletal Adverse Events (AI-related MS-AEs).	Personalized therapies targeting the OPG/TRAIL pathway for carriers of this Minor C allele may reduce cancer progression.	Geerts et al. [14], Wang et al. [25]
*rs2073617*	Major T allele increases breast cancer frequency; the CC minor allele is protective.	SNP-based stratification could guide therapies in high-risk groups.	Geerts et al. [14]
*rs34945627*	Reduces disease-free survival and overall survival in breast cancer patients.	SNP profiling may help predict prognosis and tailor aggressive treatments for affected individuals.	Ferreira et al. [24]
*rs7984870*	This SNP of RANKL has been associated with Aromatase Inhibitor-Related Musculoskeletal Adverse Events (AI-related MS-AEs).	Incorporating SNP testing can help reduce adverse events by personalizing aromatase inhibitor regimens.	Wang et al. [25]

## Data Availability

No new data were generated in the writing of this review article.

## Abbreviations

OPG	Osteoprotegerin
TNFRSF11B	Tumor Necrosis Factor Receptor Superfamily Member 11B
RANK	Receptor Activator of Nuclear Factor-κB
RANKL	Receptor Activator of Nuclear Factor-κB Ligand
TRAIL	TNF-Related Apoptosis-Inducing Ligand
SNP	Single Nucleotide Polymorphism
BRCA	Breast Cancer Gene
NF-κB	Nuclear Factor Kappa-Light-Chain-Enhancer of Activated B Cells
TNF	Tumor Necrosis Factor
DR	Death Receptor (e.g., DR4 & DR5)
PTHrP	Parathyroid Hormone-Related Protein
M-CSF	Macrophage Colony-Stimulating Factor
IL	Interleukin (e.g., IL-1α, IL-6, IL-8, IL-1B)
EPIC	European Prospective Investigation into Cancer and Nutrition
ER	Estrogen Receptor (e.g., ER-positive or ER-negative)
TNBC	Triple-Negative Breast Cancer
MMP-2	Matrix Metalloproteinase-2
AI	Aromatase Inhibitor
MS-AEs	Musculoskeletal Adverse Events
HMEC	Human Mammary Epithelial Cells
HMVEC-d	Human Microvascular Endothelial Cells (dermal)
MAPK	Mitogen-Activated Protein Kinase
PI	Propidium Iodide
IAK-1	Increase-in-Ploidy and Aurora-related Kinase 1
Bub1	Budding Uninhibited by Benzimidazoles 1
BubR1	Budding Uninhibited by Benzimidazoles-Related 1
BRCA1	Breast Cancer 1 (Gene)
BRCA2	Breast Cancer 2 (Gene)
HRT	Hormone Replacement Therapy
WNT	Wingless-related Integration Site
EMT	Epithelial-Mesenchymal Transition
STAT3	Signal Transducer and Activator of Transcription 3
rOPG	Recombinant Osteoprotegerin
OS	Overall Survival
DFS	Disease-Free Survival
ER−	Estrogen Receptor Negative
ER+	Estrogen Receptor Positive
PAH	Pulmonary Arterial Hypertension
VEGF	Vascular Endothelial Growth Factor
